# 

**Published:** 1897-09

**Authors:** 


SEPTEMBER, 1897.

*Vol. XV.* *No.* **57.**

LIBRARY OF CONGRESS
OCT 20 1897
271965
SMITHSONIAN DEPOSIT

### THE BRISTOL MEDICO-CHIRURGICAL JOURNAL

**A Quarterly Journal of the Medical Sciences for the West of England and South Wales**

*PUBLISHED UNDER THE AUSPICES OF*
*THE BRISTOL MEDICO-CHIRURGICAL SOCIETY.*

"Scire est nescire, nisi id me

Scire alius sciret."

*Editor:* R. SHINGLETON SMITH, M.D., B.Sc.

WITH WHOM ARE ASSOCIATED

J. MICHELL CLARKE, M.A., M.D.
F. R. CROSS, M.B. AND W. H. HARSANT.

*Assistant-Editor:* L. M. GRIFFITHS.

*Editorial Secretary:* B. M. H. ROGERS, B.A., M.D., B.Ch.

BRISTOL: J. W. ARROWSMITH;
LONDON: J. & A. CHURCHILL, 7 GREAT MARLBOROUGH STREET.

*Price One Shilling and Sixpence.*

